# Patterns of Anti-Inflammatory and Immunomodulating Drug Usage and Microvascular Endothelial Function in Rheumatoid Arthritis

**DOI:** 10.3389/fcvm.2021.681327

**Published:** 2021-07-19

**Authors:** Arduino A. Mangoni, Richard J. Woodman, Matteo Piga, Alberto Cauli, Anna Laura Fedele, Elisa Gremese, Gian Luca Erre, The EDRA Study Group, Floriana Castagna

**Affiliations:** ^1^Discipline of Clinical Pharmacology, Flinders Medical Centre, College of Medicine and Public Health, Flinders University, Adelaide, SA, Australia; ^2^Centre of Epidemiology and Biostatistics, College of Medicine and Public Health, Flinders University, Adelaide, SA, Australia; ^3^Dipartimento di Scienze Mediche e Sanità Pubblica, Università degli Studi di Cagliari, Cagliari, Italy; ^4^Unità Operativa Complessa (UOC) di Reumatologia, Policlinico Universitario Azienda Ospedaliero-Universitaria (AOU) Cagliari, Cagliari, Italy; ^5^Fondazione Policlinico Gemelli-Istituto di Ricovero e Cura a Carattere Scientifico (IRCCS), Università Cattolica del Sacro Cuore, Rome, Italy; ^6^Dipartimento di Scienze Mediche, Chirurgiche e Sperimentali, Università degli Studi di Sassari, Sassari, Italy; ^7^Dipartimento di Specialità Mediche, Unità Operativa Complessa (UOC) Reumatologia, Azienda Ospedaliero-Universitaria di Sassari, Sassari, Italy

**Keywords:** latent class analysis, rheumatoid arthritis, endothelial dysfunction, hydroxychloroquine, TNF-inhibitors, immunomodulating drugs, anti-inflammatory drugs

## Abstract

**Objectives:** Specific anti-inflammatory and/or immunomodulating drugs (AIDs) can influence endothelial function which is often impaired in patients with rheumatoid arthritis (RA). We sought to determine whether overall patterns of AID usage are similarly associated with endothelial function.

**Methods:** The reactive hyperaemia index (RHI), a marker of microvascular endothelial function, was measured in 868 RA patients reporting their intake of seven AIDs known to affect endothelial function. Latent class analysis (LCA) was performed to characterise patterns of AID usage. Models for 2–6 classes were compared using the AIC and BIC statistics and Lo-Mendell-Rubin likelihood ratio tests. Associations between the classes and RHI were adjusted for age, gender, body mass index, diabetes, HDL-cholesterol, LDL-cholesterol, family history of ischaemic heart disease, smoking status, RA duration, DAS28 score, steroid dose, existing hypertension, and C-reactive protein.

**Results:** LCA identified five distinct AID usage classes: Class 1, generally low medication usage; Class 2, using either sulfasalazine or non-tumour necrosis factor (TNF) inhibitors; Class 3, methotrexate users; Class 4, TNF-inhibitor users; and Class 5, hydroxychloroquine users. The geometric mean for the RHI for subjects in classes 1 to 5 was 1.92, 1.81, 1.94, 2.10, and 2.07, respectively, with subjects in classes 4 and 5 having better endothelial function than subjects in class 2 (*p* = 0.003 for each). The glucocorticoid dosage did not influence the classes formed or the association between the classes and the RHI in sensitivity analyses.

**Conclusion:** There were five broad patterns (classes) of AID usage in RA patients. The RHI was relatively lower in users of either sulfasalazine or non-TNF inhibitors. TNF inhibitors or hydroxychloroquine may counteract the negative effects of RA on endothelial function.

## Introduction

Endothelial dysfunction, affecting both the macrovascular and the microvascular beds, is commonly observed in patients with rheumatoid arthritis (RA), even in the absence of overt atherosclerotic cardiovascular disease (1–4). In particular, microvascular endothelial dysfunction might adversely affect local blood flow regulation, e.g., in the coronary circulation, with a consequent increased risk of ischaemic events (5). Notably, measures of microvascular, but not macrovascular, endothelial dysfunction have been shown to provide additional predictive capacity towards cardiovascular events to that of established risk scoring systems (6, 7). The exact mechanisms responsible for the onset and the progression of microvascular endothelial dysfunction in RA are largely unknown, although the presence of a pro-inflammatory and pro-oxidant state seems to play an important role (3, 8, 9). Several drugs with anti-inflammatory and/or immunomodulating effects (AIDs), e.g., non-steroidal inflammatory drugs (NSAIDs), glucocorticoids and disease-modifying anti-rheumatic drugs (DMARDs, synthetics, targeted synthetics, and biologics) are commonly prescribed in RA patients to control disease activity and mitigate the various intra- and extra-articular clinical manifestations (10). A number of studies have investigated the effects of different AIDs, particularly the DMARDs methotrexate and several biologics and glucocorticoids, on endothelial function in RA (1). However, the interpretation of the data on the association between the use of these drugs and measures of endothelial function is difficult as participants were generally treated with additional AIDs. As most patients with RA are routinely managed with different combinations of NSAIDs, glucocorticoids, and DMARDs assessing the effects of patterns of AID usage, rather than individual drugs, might be particularly useful for the identification of pharmacological strategies, including combinations of AIDs, exerting beneficial effects on endothelial function and, potentially, cardiovascular risk in RA.

Latent class analysis (LCA), is an objective (model-based) unsupervised machine learning technique that enables the identification of underlying classes (e.g., AID usage classes), with class membership defined by virtue of having similar values across a set of observed categorical variables (11). LCA can also be thought of as being a “person-centred” approach to analysis, with the focus of the analysis being on identifying groups of individuals with a pattern of similar values for a set of variables, in contrast to regression-based models that identify mean effects of individual variables. LCA thus captures the homogeneity of values for a set of variables within groups, and the heterogeneity of the same variable values between groups. At the same time, formal measures of model fit can be used to identify the most parsimonious number of distinct patterns (and thus class membership) in the data (12).

In this study, we used LCA to identify and describe the unique patterns of endothelial function related AID usage within a representative sample of RA patients. We then examined which, if any of these patterns were associated with better or worse microvascular endothelial function with adjustment for potential clinical and demographic confounders.

## Methods

### Study Design

We conducted a cross-sectional study examining the association between AID usage and microvascular endothelial function in patients with RA. LCA was used to determine the major patterns of AID usage. LCA is a particular form of unsupervised machine learning with unlabelled data in which the hidden labels (i.e., class membership) are derived from a set of categorical observed variables (11). Thus, the analysis clusters subjects into a smaller number of labelled classes. Our primary interest was to determine the strength of the relationship between these groups (defined by different patterns of AID usage) and measured microvascular endothelial function. Each AID was measured as a binary (yes/no) variable.

### Patient Recruitment and Ethical Approval

We studied a consecutive series of patients with RA without clinically overt atherosclerotic cardiovascular disease, as part of the multicentre (three Italian hospital sites: Sassari, Cagliari, and Rome) Endothelial Dysfunction Evaluation for Coronary Heart Disease Risk Estimation in Rheumatoid Arthritis (EDRA) study (ClinicalTrials.gov: NCT02341066) between October 2015 and July 2017. The EDRA study was approved by the Azienda ASL 1 of Sassari (Italy) Institutional Review Board (2126/CE-2015) and was conducted in accordance with the Declaration of Helsinki.

Each subject signed a written informed consent before participation. Inclusion criteria were: 1) men and women aged >45 and <84 years, and 2) RA as defined by the ACR/EULAR 2010 RA classification criteria (13). Exclusion criteria were: 1) previous cardiovascular or cerebrovascular events, e.g., acute coronary syndrome, stable angina, stroke, interventional procedures, carotid endarterectomy, and symptomatic peripheral artery ischaemia, 2) abnormal electrocardiogram at rest, 3) signs or symptoms of autonomic nervous system dysfunction, 4) severe infections in the previous six months, 5) overt liver failure and/or renal disease (GFR <30 mL/min, Cockroft-Gault formula), 6) recent diagnosis of cancer, and 7) pregnancy.

### Microvascular Endothelial Function

Microvascular endothelial function was assessed by measuring the reactive hyperaemia of the small digital artery (reactive hyperaemia index, RHI) after an ischaemic stimulus using pulse amplitude tonometry (PAT). PAT has been shown to be significantly associated with measures of coronary microvascular endothelial function (14), cardiovascular risk factors (15), and cardiovascular events (16). Patients were studied in a fasting state. Antihypertensive drugs were withheld on the study day. Finger probes, consisting of thimble a-shaped sensor cap which register pulsatile volume changes, were placed on the middle finger of each hand. Changes in digital pulse amplitude were detected by pressure transducers, filtered, amplified, and then recorded for further analysis by the EndoPAT 2000 device (Itamar Medical Inc., Caesarea, Israel). After a 5 min baseline measurement, the brachial artery flow was interrupted by a cuff placed on the proximal forearm and inflated to 200 or 60 mmHg above the systolic blood pressure, for 5 min. Then, the cuff was deflated, and the digital pulse amplitude was recorded for a further 6 min. The ratio of the post-ischaemic pulse amplitude signal compared with baseline was calculated, normalised for the baseline signal, and indexed to the contralateral one. The log-transformed ratio, expressed as Ln-RHI, reflected the small artery reactive hyperaemia, with lower values representing impaired endothelial function.

### Baseline Characteristics and AID Usage

We collected data on hypertension (blood pressure ≥140/90 mmHg or treatment with antihypertensive drugs), diabetes mellitus (patient history and/or treatment with insulin or oral hypoglycaemic agents), family history of ischaemic heart disease (IHD) in first-degree relatives, smoking habit, total, HDL- and LDL-cholesterol, triglycerides, steroid treatment and cumulative steroid dose in the last month, number of swollen joints, number of tender joints, C-reactive protein (CRP), erythrocyte sedimentation rate (ESR), Disease Activity Score-28 (DAS-28), Health Assessment Questionnaire (HAQ), positivity for IgM-rheumatoid factor (IgM-RF), and anti-citrullinated cyclic peptide antibodies (ACPA). The specific seven AIDs of interest that were used for the LCA included NSAIDs and the DMARDs methotrexate, leflunomide, hydroxychloroquine, sulfasalazine, and the biologics TNF-inhibitors and non-TNF inhibitors. These agents were selected as they have been previously shown to influence endothelial function in experimental and clinical studies of RA and other disease states (1, 17–22).

### Statistical Analysis

Descriptive statistics were used to describe the baseline characteristics of RA patients using either mean and standard deviation for normally distributed continuous variables, median and inter-quartile range for non-normally distributed continuous variables and frequency (percentage) for categorical variables. Correlations between the endothelial related AIDs were estimated using Spearman's rho correlation coefficient. LCA was performed to identify the pattern of medication usage of the seven AIDs of interest. Models were estimated for between 2 and 6 latent classes, and for each model the class membership of each subject was decided based their highest (posterior) predicted probability of class membership. Model selection was based on several model fit statistics including the Akaike Information Criterion (AIC), the Bayesian Information Criterion (BIC) (smaller values are better for each), and entropy (larger values are better). Likelihood ratio tests were also used to assess the difference in fit between models. The final selected model was based on both model fit (lowest AIC and BIC and largest entropy) and consideration of the number of subjects assigned to each class with a minimum of 5% of all subjects in a single class used as a rule of thumb for the smallest class size (23). Line plots of the proportion of medication usage for each of the seven AIDs were used to identify the main medications characterising each class. We compared the Ln-RHI values across latent classes using multivariate linear regression with adjustment for age and gender (Model 1) and additional adjustment for body mass index (BMI), diabetes (yes/no), HDL-cholesterol, LDL-cholesterol, family history of IHD, smoking status (never, light, moderate, severe, or Former), RA duration, DAS28 score, steroid dose (mg/day), hypertension (yes/no), and CRP (Model 2). In each of these models we assessed the overall significance of the class membership variable on endothelial function using a Wald test and differences between classes on endothelial function using a *z*-score test. Due to the relatively high number of missing values for some variables, particularly smoking status, each regression analysis was performed after using multiple imputation using Stata's predictive mean matching algorithm with five nearest neighbours. The variables to be imputed and also used for imputation themselves included age, BMI, HDL-cholesterol, LDL-cholesterol, diabetes, smoking status, family history of IHD, RA duration, DAS28 score, CRP, and steroid dosage. Twenty sets of data were imputed, the analysis was performed on the imputed datasets and model estimates for each regression were then combined using standard methods. Finally, we assessed the independent associations between RHI values and each individual AID using a standard multivariate regression model approach with the seven AIDs included as exposure variables and the same adjustment variables as used in the LCA regression models. The LCA was performed using Mplus software. STATA software (StataCorp, version 15.1, USA) was used for the regression analyses and descriptive statistics. In Mplus, the LCA model was defined using the seven medication variables with each medication variable declared as being categorical and the Analysis type = mixture option was used to define the LCA model. An overall 2-sided type 1 error rate of α = 0.05 was considered statistically significant for determining differences across classes in all regression analyses.

## Results

### Patient Recruitment and Missing Data

A total of 874 patients were recruited and 868 provided complete information on endothelial function related AIDs, were tested for microvascular endothelial function, and were therefore included in the analysis (Table 1). The amount of missing data for covariates for the 868 patients were: age (*n* = 8 missing values), HDL-cholesterol (*n* = 61), LDL cholesterol (*n* = 70), diabetes status (*n* = 7), smoking status (*n* = 9), family history of IHD (*n* = 10), RA duration (*n* = 9), DAS28 (*n* = 31), CRP (*n* = 24), steroid dosage (*n* = 2), and BMI (*n* = 16). Of the 868 patients, 748 had no missing data and 120 had missing data for at least one covariate. Data was imputed for all missing values for 20 separate datasets and were combined with the results of the LCA for regression analysis with the MI datasets. TNF inhibitors (*n* = 244) used included etanercept (*n* = 131), infliximab (*n* = 4), adalimumab (*n* = 82), golimumab (*n* = 9), and certolizumab (*n* = 18). Non-TNF inhibitors (*n* = 127) used included abatacept (*n* = 44), tocilizumab (*n* = 62), and rituximab (*n* = 21).

**Table 1:** Baseline clinical characteristics of the study population (*n* = 868).

	**All subjects (*n* = 868)**	**Class 1 (*n* = 156)**	**Class 2 (*n* = 58)**	**Class 3 (*n* = 452)**	**Class 4 (*n* = 91)**	**Class 5 (*n* = 111)**	***p*-value^a^**
Age (years), mean ± SD	60.9 ± 9.4	60.3 ± 10.2	59.7 ± 8.2	61.4 ± 9.45	60.0 ± 8.9	61.2 ± 9.0	0.446
Female, *n* (%)	656 (75.6)	123 (78.9)	49 (84.5)	334 (73.9)	68 (74.7)	82 (73.9)	0.367
BMI (kg/m^2^), median(IQR)	25.3 (22.7, 28.1)	25.0 (23.0, 27.6)	25.0 (21.8, 26.8)	25.4 (22.7, 28.3)	24.4 (22.1, 27.6)	25.5 (22.8, 28.6)	0.319
HDL, mean ± SD	61.3 ± 16.0	60.4 ± 16.8	65.2 ± 15.3	61.2 ± 15.8	60.6 ± 17.0	61.7 ± 14.9	0.380
LDL, mean ± SD	124.4 ± 31.6	124.5 ± 35.8	133.9 ± 26.6	124.6 ± 30.7	130.3 = 32.8	114.4 ± 28.6	<0.001
Disease duration (months), median (IQR)	101 (48, 180)	99 (36, 192)	133 (76, 207)	90 (48, 165)	156 (108, 216)	84 (48, 170)	<0.001
DAS28, mean ± SD	3.52 ± 1.35	3.96 ± 1.44	3.50 ± 1.26	3.40 ± 1.35	3.41 ± 1.09	3.54 ± 1.36	<0.001
Steroid dose (mg/day)	0 (0, 5)	2.5 (0, 5)	1.25 (0, 5)	0 (0, 2.5)	0 (0, 0)	0 (0, 2.5)	<0.001
C-reactive protein (mg/L), median (IQR)	0.30 (0.11, 0.70)	0.30 (0.12, 0.71)	0.34 (0.13, 0.73)	0.30 (0.10, 0.70)	0.26 (0.11, 0.79)	0.31 (0.14, 0.67)	0.778
Clinic SBP (mmHg), mean ± SD	128 ± 17	127 ± 16	126 ± 16	128 ± 17	129 ± 17	129 ± 16	0.785
Clinic DBP (mmHg), mean ± SD	77 ± 10	77 ± 9	78 ± 9	77 ± 10	78 ± 10	76 ± 9	0.743
Diabetes, *n* (%)	64 (7.4)	9 (5.8)	8 (13.8)	36 (8.0)	3 (3.3)	8 (7.3)	0.183
Family history CVD, *n* (%)	263 (30.6)	37 (24.0)	23 (39.7)	147 (32.9)	26 (28.9)	30 (27.5)	0.132
**Smoking status**, ***n*** **(%)**							
Never	407 (47.4)	69 (46.3)	24 (41.4)	217 (48.4)	45 (50.0)	50 (46.3)	0.009
Light	68 (7.9)	15 (10.1)	8 (13.8)	28 (6.3)	8 (8.9)	8 (7.4)	
Moderate	82 (9.6)	12 (8.1)	28 (6.3)	50 (11.2)	6 (5.6)	6 (5.6)	
Severe	30 (3.5)	1 (0.7)	8 (8.9)	14 (3.1)	8 (7.4)	8 (7.4)	
Former	272 (31.7)	52 (34.9)	8 (7.4)	139 (31.0)	36 (33.3)	36 (33.3)	
Hypertension, *n* (%)	453 (52.2)	87 (55.8)	30 (51.7)	230 (50.9)	51 (56.0)	55 (49.6)	0.742
Use of hypertensive drugs, *n* (%)	312 (36.2)	64 (41.3)	19 (32.8)	158 (35.2)	32 (35.6)	39 (35.8)	0.688
NSAIDS, *n* (%)	203 (23.4)	56 (35.9)	14 (24.1)	114 (23.3)	0 (0.0)	19 (17.1)	<0.001
Methotrexate use, *n* (%)	546 (62.9)	0 (0.0)	36 (62.1)	452 100.0)	0 (0.0)	58 (52.3)	<0.001
Leflunomide use, *n* (%)	73 (8.4)	46 (29.5)	0 (0.0)	5 (1.1)	22 (24.2)	0 (0.0)	<0.001
Hydroxychloroquine use, *n* (%)	131 (15.1)	15 (9.6)	0 (0.0)	5 (1.1)	0 (0.0)	111 (100.0)	<0.001
Sulfasalazine use, *n* (%)	35 (4.0)	1 (0.6)	24 (41.4)	0 (0.0)	4 (4.4)	6 (5.4)	<0.001
TNF inhibitor use, *n* (%)	244 (28.1)	22 (14.1)	0 (0.0)	135 (29.9)	87 (95.6)	0 (0.0)	<0.001
Non-TNF inhibitor use, *n* (%)	127 (14.6)	35 (22.4)	44 (75.9)	39 (8.6)	1 (1.1)	8 (7.2)	<0.001
Non-TNF/Sulfasalazine use, *n* (%)	150 (17.3)	35 (22.4)	58 (100.0)	39 (8.6)	5 (5.5)	13 (11.7)	<0.001
Loge RHI, mean ± SD	0.67 ± 0.33	0.65 ± 0.33	0.59 ± 0.30	0.66 ± 0.31	0.74 ± 0.39	0.73 ± 0.32	0.014
RHI, geometric mean (95% CI)	1.96 (1.92, 2.00)	1.92 (1.82, 2.03)	1.81 (1.67, 1.95)	1.94 (1.88, 2.00)	2.10 (1.94, 2.27)	2.07 (1.95, 2.20)	0.004

a *P-value for difference between profiles using ANOVA (normal distributions), test of medians (asymmetric distributions), Chi-squared test, or Fishers Exact (categorical)*.

### Latent Class Analysis

All LCA analyses with specified models for 2, 3, 4, 5, and 6 classes converged successfully and the AIC and BIC statistics and *p*-values for the Lo-Mendell-Rubin tests are described in Table 2. Based on the sample size adjusted BIC, the entropy, likelihood ratio tests for 4 vs. 5 classes, and 5 vs. 6 classes, the optimal number of classes was chosen as being 5. These 5 classes each included between 6.7 and 52.1% of the original sample size (Table 2). The mean probability of accurately assigned profile membership ranged from 0.43 for patients in class 2 to 1.00 for those in class 5 (Table 3) indicating an overall high degree of certainty that everyone was assigned to the correct medication usage class.

**Table 2:** Summary of fit statistics for latent class models.

**Classes in model**	**AIC**	**BIC**	**SSABIC**	**Entropy**	**LMR ALRT *p*-value** **(k vs. k-1 classes)**	**VLMR *p*-value** **(k vs. k-1 classes)**	**PBLRT *p*-value** **(k vs. k-1 classes)**	**% in each class (in order of size)**					
								**Class 1**	**Class 2**	**Class 3**	**Class 4**	**Class 5**	**Class 6**
1	5,022	5,055	5,033	NA	NA	NA	NA	100.0					
2	4,900	4,971	4,923	1.000	<0.001	<0.001	<0.001	62.9	37.1				
3	4,861	4,971	4,898	0.646	0.0002	0.0002	<0.001	41.1	35.1	23.8			
4	4,843	4,991	4,892	0.797	0.0002	0.0002	<0.001	48.2	27.5	13.7	10.6		
5	4,843	5,029	4,905	0.778	0.0299	0.0279	0.079	52.1	18.0	12.8	10.5	6.7	
6	4,848	5,072	4,923	0.703	0.0189	0.0178	0.4286	50.9	14.4	11.9	10.6	7.0	5.2

*AIC, Akaike information criterion; BIC, Bayesian information criterion; SSABIC, Sample size adjusted BIC; LMR ALRT, Lo-Mendell-Rubin Adjusted LR test; VLMR, Vuong-Lo-Mendell-Rubin likelihood ratio test; PBLRT, Parametric Bootstrapped Likelihood Ratio Test*.

**Table 3:** Mean posterior probabilities associated with class membership in the 5-class LCA model.

	***n***	**%**	**Probability of membership in each class**				
**Assigned class**			**Class 1**	**Class 2**	**Class 3**	**Class 4**	**Class 5**
Class 1	156	18.0	0.738	0.023	0.000	0.157	0.082
Class 2	58	6.7	0.180	0.432	0.388	0.000	0.000
Class 3	452	52.1	0.000	0.004	0.971	0.000	0.024
Class 4	91	10.5	0.005	0.000	0.000	0.995	0.000
Class 5	111	12.8	0.000	0.000	0.000	0.000	1.000

### Qualitative Labelling of the Latent Classes

**Figure 1:**
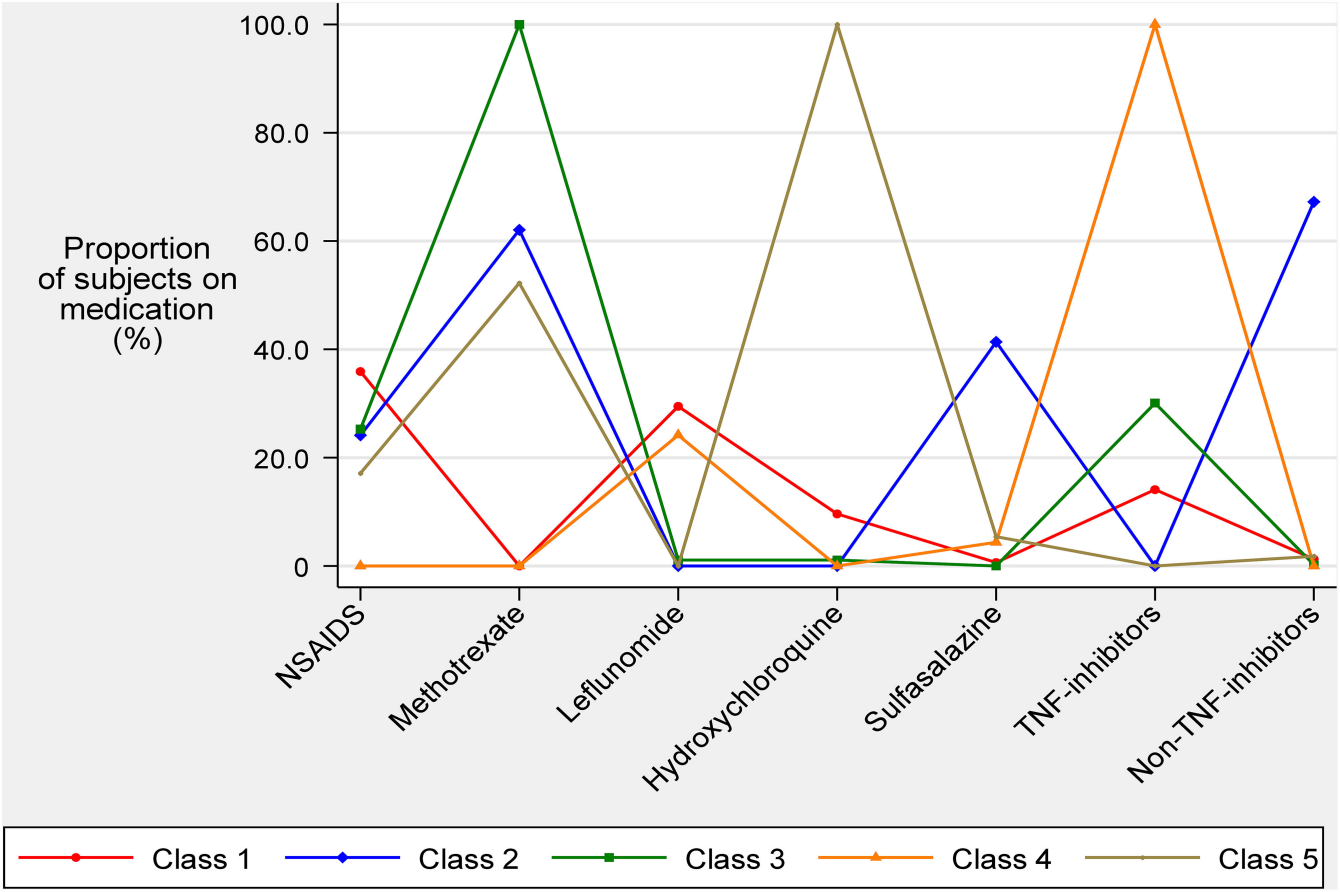
Average medication usage of anti-inflammatory and immunomodulating drugs known to affect endothelial function for each latent class.

Figure 1 describes the mean percentage of subjects within each class using each of the seven AIDs. Class 1 (n = 156) was depicted by subjects with low overall drug usage except for NSAIDs (35.9%) and leflunomide (29.5%). Class 2 subjects had a high usage of methotrexate (62.0%), sulfasalazine (41.3%), and non-TNF inhibitors (67.2%). Class 3 subjects were characterised by the use of methotrexate (100%) with some subjects also using NSAIDs (25.3%) and/or TNF-inhibitors (30.1%). Class 4 subjects were characterised by the use of TNF-inhibitors (100%) with some subjects also using leflunomide (24.2%). Class 5 subjects were characterised by the use of hydroxychloroquine (100%) with some subjects also using methotrexate (52.2%). These statistics suggested labels for the 5 classes as follows: Class 1, low medications; Class 2, sulfasalazine and non-TNF inhibitor; Class 3, methotrexate; Class 4, TNF-inhibitors; and Class 5, hydroxychloroquine.

### Clinical and Demographic Characteristics and Medication Usage

The baseline characteristics of the 868 patients are described in Table 1. The mean ± SD age was 60.9 ± 9.4 years and 656 (75.6%) were female. There were no differences in age or gender distribution across the five latent classes. However, there were significant differences across the five classes for LDL-cholesterol, RA duration, DAS28 (*p* < 0.001 for each) and smoking status (*p* = 0.009). There were also significant differences across classes for all seven AIDs used in the LCA (*p* < 0.001 for each) and the Ln-RHI (*p* = 0.014). The Spearman's rho correlation coefficients for the seven endothelial function related AIDs were generally weak (absolute value ≤0.30) except for a moderate inverse correlation between leflunomide and methotrexate usage (ρ = −0.352; Table 4).

**Table 4:** Proportion of medication usage and Spearman's rho correlations for the 7 medications used in the LCA (*n* = 868).

	**Medication**	***N* users (%)**	**1**	**2**	**3**	**4**	**5**	**6**	**7**
1	NSAIDs	203 (23.4)	1.00						
2	Methotrexate	546 (62.9)	0.025	1.00					
3	Leflunomide	73 (8.4)	−0.050	−0.352	1.00				
4	Hydroxychloroquine	131 (15.1)	−0.036	−0.129	−0.047	1.00			
5	Sulfasalazine	35 (4.0)	−0.003	−0.036	−0.041	0.011	1.00		
6	TNF inhibitor	249 (28.7)	−0.008	−0.108	0.046	−0.168	−0.078	1.00	
7	Non-TNF inhibitors	44 (5.1)	−0.029	−0.018	−0.051	−0.068	0.086	−0.124	1.00

### Multivariate Regression Analysis of Ln-RHI on Latent Classes

Following multiple imputation, all 868 patients were included in the multivariate linear regression analysis for Ln-RHI (Table 5). After adjustment for age and gender (Model 1), there was a significant association between class membership and Ln-RHI (*p* = 0.014). Overall, class 4 and class 5 subjects had relatively higher RHI values compared to patients in class 2 (17% higher, *p* = 0.005, and 15% higher, *p* = 0.007, respectively). Following complete adjustment (Model 2), including the daily steroid dose, the relatively higher values of RHI for class 4 and class 5 compared to class 2 remained (19% higher, *p* = 0.003, and 17%, *p* = 0.003, respectively).

**Table 5:** Multivariate linear regression analysis of log transformed RHI on latent class membership (*n* = 868)^a^.

	**Model 1^b^** **Exp^β^ (95% CI)**	***p*-value^a^**	**Model 2^c^** **Exp^β^ (95% CI)**	***p*-value^a^**
Latent class		0.014^d^		0.007^d^
Class 1 (*n* = 156)	1.07 (0.97, 1.18)	0.193	1.08 (0.98, 1.19)	0.113
Class 2 (*n* = 58)	Reference	–	Reference	–
Class 3 (*n* = 452)	1.08 (0.99, 1.18)	0.087	1.09 (1.00, 1.20)	0.051
Class 4 (*n* = 91)	1.17 (1.05, 1.30)	0.005	1.19 (1.06, 1.32)	0.003
Class 5 (*n* = 111)	1.15 (1.04, 1.28)	0.007	1.17 (1.06, 1.30)	0.003

a *Using multiple imputaion (MI) with n = 598 complete observations and n = 270 non-complete observations across covariates in model 2. MI was perfromed using chained equations and the KNN predictive mean-matching algorithm*.

b *Model 1: adjusted for age and gender*.

c *Model 2: adjusted for age, gender, BMI, diabetes, HDL-cholesterol, LDL-cholesterol, family history of CHD, smoking status (Never, Light, Moderate, Severe or Former), duration of RA, DAS28 score, steroid dose (mg/day), Hypertension, and CRP*.

d *Overall p-value for latent class variable*.

### Multilevel Regression for Ln-RHI on Individual Endothelial Function Related AIDs

Table 6 shows the results of the multivariate linear regression analysis of Ln-RHI on the seven individual AIDs. In Model 1 (age and gender adjusted), sulfasalazine use was associated with a 12% lower Ln-RHI (exp^β^ = 0.88, 95% CI 0.78, 0.88, *p* = 0.02). In Model 2 (full adjustment), including daily steroid dose, sulfasalazine use was associated with an 11% lower Ln-RHI (exp^β^ = 0.89, 95% CI 0.80, 0.99, *p* = 0.032) and hydroxychloroquine was associated with a 7% higher Ln-RHI (exp^β^ = 1.07, 95% CI 1.00, 1.14, *p* = 0.044).

**Table 6:** Multivariate linear regression analysis of log transformed RHI on the 7 individual endothelial related drugs (*n* = 868)^a^.

	**Model 1^b^** **Exp^β^ (95% CI)**	***p*-value**	**Model 2^c^** **Exp^β^ (95% CI)**	***p*-value**
NSAIDS	0.98 (0.93, 1.02)	0.366	0.98 (0.93, 1.03)	0.424
Methotrexate	0.97 (0.93, 1.02)	0.295	0.98 (0.93, 1.03)	0.346
Leflunomide	0.96 (0.88, 1.05)	0.367	0.97 (0.89, 1.05)	0.404
Hydroxychloroquine	1.06 (0.99, 1.12)	0.086	1.07 (1.00, 1.14)	0.044
Sulfasalazine	0.88 (0.78, 0.98)	0.020	0.89 (0.80, 0.99)	0.032
TNF inhibitor	0.99 (0.94, 1.04)	0.574	1.00 (0.94, 1.05)	0.784
Non-TNF inhibitors	0.93 (0.84, 1.03)	0.167	0.91 (0.83, 1.01)	0.084

a *Using multiple imputaion (MI) with n = 598 complete observations and n = 270 non-complete observations across covariates in models 1 and 2. MI was perfromed using chained equations and the KNN predictive mean-matching algorithm*.

b *Model 1: All 7 drugs and adjusted for age and gender*.

c *Model 2: All 7 drugs and adjusted for age, gender, BMI, diabetes, HDL-cholesterol, LDL-cholesterol, family history of CHD, smoking status (Never, Light, Moderate, Severe, or Former), duration of RA, DAS28 score, steroid dose (mg/day), Hypertension, and CRP*.

## Discussion

In this study we used LCA, an unsupervised machine learning technique, to identify five broad usage patterns of AIDs within a population of patients with RA. Following classification of the RA subjects into one of the five classes, we used regression analysis to show that the five classes were associated with measures of microvascular endothelial function. Patients in class 4, ninety-six percent of whom used TNF-inhibitors, and patients in class 5 that all used hydroxychloroquine, had significantly better microvascular endothelial function than those patients in class 2 who all used either sulfasalazine or non-TNF inhibitors. Of note, only a small percentage of subjects in classes 4 and 5 used either sulfasalazine or non-TNF inhibitors (5.5 and 11.7%, respectively). Patients in class 1 and class 3 who did generally not use TNF inhibitors or hydroxychloroquine did not have lower endothelial function compared to subjects in class 2. In addition, multivariate regression using the seven individual AIDs as exposure variables instead of class membership demonstrated an independent protective effect of hydroxychloroquine on microvascular endothelial function.

A number of experimental and clinical studies have investigated the effects of different AIDs on measures of endothelial function and synthesis of nitric oxide (NO), a key endogenous messenger that is synthesised in the endothelial cells and mediates several effects on vascular homeostasis, e.g., vasodilatation, smooth muscle cell proliferation, and modulation of arterial stiffness (24). The AIDs that have been particularly studied in the context of RA include the glucocorticoids and the DMARDs methotrexate and several biologics, i.e., TNF and non-TNF inhibitors (1). Studies investigating methotrexate have reported contrasting effects on endothelial function. Differences in background therapy with other DMARD or non-DMARD agents, baseline endothelial function, RA disease activity, and treatment duration might explain, at least in part, such discrepancies (1). By contrast, a systematic review and meta-analysis has shown that treatment with TNF inhibitors is associated with a significant improvement in endothelial function in patients with RA (22). Similarly, four small studies have reported beneficial effects of non-TNF inhibitor biologics, i.e., tocilizumab, anakinra, and rituximab, on endothelial function in RA patients with or without background treatment with other DMARDs (25–28). Finally, treatment with glucocorticoids has been shown to exert either neutral or beneficial effects on endothelial function, again in RA patients already receiving background therapy with DMARDs (29, 30).

Our study, based on a different statistical approach that consisted in assessing patterns of AID usage, rather than of individual AIDs, suggests that the use of hydroxychloroquine or TNF-inhibitors is associated with better microvascular endothelial function when compared to the usage of sulfasalazine or non-TNF inhibitors in RA patients. Several lines of evidence suggest that hydroxychloroquine might exert protective effects on endothelial function and vascular homeostasis both *in vitro* and *in vivo*. For example, hydroxychloroquine has shown atheroprotective effects by targeting toll-like receptor signalling, cytokine synthesis, activation of T-cells and monocytes, oxidative stress pathways, and endothelial dysfunction, in systemic lupus erythematosus and experimental models of inflammation and pre-eclampsia (31–33). The beneficial effects of TNF inhibitors on endothelial function previously reported can primarily be attributed to their main pharmacodynamic effect (22). There is good evidence that the cytokine TNF-α exerts significant detrimental effects on the structural and functional integrity of the endothelium, primarily through the increased local synthesis of reactive oxygen species (34). Little is known on the effects of sulfasalazine on endothelial function and vascular homeostasis. A study in patients with IHD reported that acute, 4-day, treatment with sulfasalazine did not exert any significant effect on microvascular or macrovascular endothelial function (35). By contrast, in an animal model of sickle cell disease, sulfasalazine treatment significantly improved microvascular blood flow (36). Whilst, as previously discussed, the non-TNF inhibitor biologics tocilizumab and anakinra have shown beneficial effects on endothelial function in RA (25, 26), this association was not confirmed in our study. It should be emphasised that a fraction of our patients was on tocilizumab whereas the remaining were prescribed abatacept and rituximab. A recent systematic review has identified studies investigating the effects of abatacept (*n* = 2) and rituximab (*n* = 8) on measures of vascular function in patients with RA. However, the studies on abatacept did not assess microvascular or macrovascular endothelial function whereas four studies on rituximab investigated macrovascular (flow-mediated brachial artery dilatation), but not microvascular, endothelial function. Three of these studies reported an improvement in flow-mediated dilatation whereas the remaining one did not show any significant change with treatment (37). Further research is required to investigate the effects of abatacept and rituximab on microvascular endothelial function. However, the results of our study do not necessarily imply a negative effect of sulfasalazine and non-TNF inhibitors on microvascular endothelial function in RA, rather the presence of significant differences in Ln-RHI values between specific patterns of AID usage in favour of hydroxychloroquine and TNF-inhibitors. Treatment with hydroxychloroquine and TNF-inhibitors, singly or in combination, might confer protection against the detrimental effects of RA on endothelial function and, more generally, vascular homeostasis (1–3). The potential vasculoprotective effects of hydroxychloroquine are supported by the results of a recent systematic review and meta-analysis, which reported that the use of hydroxychloroquine and its analogue chloroquine in patients with rheumatic diseases was associated with a significant reduction in the risk of cardiovascular events (pooled risk ratio, RR, 0.72, 95% CI 0.56–0.94, *p* = 0.013) (38). Similarly, another systematic review and meta-analysis showed that, in patients with RA, treatment with TNF inhibitors was associated with a significantly lower risk of cardiovascular events (RR 0.70, 95% 0.54–0.90, *p* = 0.005) (39). The results of our study mandate the conduct of adequately designed interventional studies to investigate the effects of treatment with hydroxychloroquine, with or without TNF inhibitors, on endothelial function and overall cardiovascular risk in patients with RA. Given the reported similarities between the pathogenesis of RA and that of atherosclerosis the evidence of protective effects with hydroxychloroquine and/or TNF from such trials might translate into the possible repurposing of these drugs for cardiovascular risk management in other, non-autoimmune, patient populations.

An important limitation of our study is the cross-sectional design, which does not allow to establish a clear cause-effect relationship between the identified patterns of AID usage and the RHI. Furthermore, no information was available regarding the treatment duration for individual AIDs and the specific NSAIDs used. In particular, individual NSAIDs have shown different effects on endothelial function (17). Finally, the observed associations between AID usage and microvascular endothelial function need also to be confirmed with measures of macrovascular endothelial function, given pathophysiological and prognostic differences between the two vascular beds (1). Strengths include the sample size, our study being the largest assessing microvascular endothelial function in RA patients, the representability of the study population and the comprehensive set of clinical and demographic confounders, particularly RA duration and activity and the dose of steroids, that were adjusted for in our analyses.

In conclusion, our study suggests that specific patterns of AIDs usage, particularly hydroxychloroquine and/or TNF inhibitors, may counteract the negative effects of RA on microvascular endothelial function. Interventional studies are required to confirm our findings and to establish the potential role of these agents in cardiovascular risk management in RA and other patient groups.

## Data Availability Statement

The raw data supporting the conclusions of this article will be made available by the authors, without undue reservation.

## Ethics Statement

The studies involving human participants were reviewed and approved by Azienda ASL 1 of Sassari (Italy) Institutional Review Board (2126/CE-2015). The patients/participants provided their written informed consent to participate in this study.

## Author Contributions

GE, AM, and RW contributed to conception and design of the study. GE, MP, and AF organised the database. RW performed the statistical analysis. AM wrote the first draught of the manuscript. GE, AM, RW, EG, and AC wrote sections of the manuscript. All authors contributed to manuscript revision, read, and approved the submitted version.

## Edra Study Group Collaborators

Floriana Castagna^1^, Marco Piras^1^, Maria Luisa Cadoni^1^, Loredana Taras^1^, Ignazio Cangemi^2^, Martina Dessì^2^, Ilaria Platè^2^, Elisabetta Chessa^2^, Mattia Congia^2^, Alberto Floris^2^, Maria Giovanna Longu^1^, Giuseppe Passiu^2,3^, Dario Bruno^4^ and Gianfranco Ferraccioli^4^

^1^ Dipartimento di Scienze Mediche, Chirurgiche e Sperimentali, Università degli Studi di Sassari, Sassari, Italy

^2^ Dipartimento di Scienze Mediche e Sanità Pubblica, Università degli Studi di Cagliari, Cagliari, Italy

^3^ Dipartimento di Specialità Mediche, UOC Reumatologia, Azienda Ospedaliero-Universitaria di Sassari, Sassari, Italy

^4^ Fondazione Policlinico Gemelli-IRCCS, Università Cattolica del Sacro Cuore, Roma, Italy

## Conflict of Interest

The authors declare that the research was conducted in the absence of any commercial or financial relationships that could be construed as a potential conflict of interest.
